# The effects of ninjin’yoeito on the electrophysiological properties of dopamine neurons in the ventral tegmental area/substantia nigra pars compacta and medium spiny neurons in the nucleus accumbens

**DOI:** 10.18632/aging.204109

**Published:** 2022-06-03

**Authors:** Ryota Imai, Keita Mizuno, Yuji Omiya, Kazushige Mizoguchi, Yuko Maejima, Kenju Shimomura

**Affiliations:** 1 Kampo Research and Development Division, Tsumura & Co., Ibaraki, Japan; 2 Department of Bioregulation and Pharmacological Medicine, Fukushima Medical University School of Medicine, Fukushima, Japan

**Keywords:** reward system, nucleus accumbens, dopamine, Kampo, neuronal excitability

## Abstract

The ventral tegmental area (VTA), substantia nigra pars compacta (SNpc) and nucleus accumbens (NAc) are involved in the regulation of appetite and motivational behaviors. A traditional Japanese (Kampo) medicine, ninjin’yoeito (NYT), has been reported to improve decreased motivation and anorexia in patients with Alzheimer’s disease and apathy-like model mice. Thus, NYT may affect the activities of neurons in the VTA, SNpc and NAc. However, little is known about the underlying mechanisms of NYT. Here, we investigated the effects of NYT on the electrophysiological properties of dopaminergic neurons in the VTA and SNpc, as well as on those of medium spiny neurons (MSNs) in the NAc (core and shell subregions), by applying the patch-clamp technique in the brain slices. NYT reduced the resting membrane potential of VTA and SNpc dopaminergic neurons. In contrast, NYT increased the firing frequency of NAc MSNs accompanied by shortened first spike latency and interspike interval. Furthermore, NYT attenuated the inward rectification and sustained outward currents. In conclusion, NYT may directly influence the excitability of dopaminergic neurons in the VTA and SNpc, as well as MSNs in the NAc (core and shell). NYT may modulate dopamine signals in appetite and motivational behaviors.

## INTRODUCTION

The reward system in the brain, which is comprised of areas such as the ventral tegmental area (VTA), substantia nigra pars compacta (SNpc), nucleus accumbens (NAc), striatum, and prefrontal cortex (PFC), regulates fundamentally motivational behaviors. Among various neurotransmitters, dopamine (DA) has been investigated most intensively because it is a key neurotransmitter mediating reward-relevant neural circuitry.

Dopaminergic (DAergic) neurons residing in the VTA and SNpc regulate response to rewards, goal-directed behavior and movement [1]. DA is also considered to have a significant influence on feeding behavior, since DA-deficient mice become hypophagic, which ultimately leads to death by starvation unless L-DOPA treatment is applied [2, 3]. In addition, a previous study using rodents has shown that VTA DAergic neurons can be activated by ghrelin, an appetite-promoting peptide, which increases food intake [4]. Additionally, DAergic neuron depletion of the SNpc produces animals with a deficit in drinking and feeding [5]. Moreover, it has also been suggested that DA is released from the SNpc during feeding in humans [6]. Therefore, the DAergic neurons in the VTA and SNpc play an important role in the regulation of feeding-related motivation [7].

The accumulated evidence has shown the function of NAc regarding feeding behavior, motor locomotion, impulsivity, motivation, learning, goal-directed behavior, processing of emotion, and encoding value of rewards [8, 9]. In particular, previous studies demonstrated that the NAc is involved in feeding behavior, based on the reports that a GABA receptor antagonist injected into the NAc increases food intake in rats [7, 10–12]. In addition, since the NAc area interacts with the VTA and SNpc areas [7], the NAc can play a key role in the DAergic pathway. Indeed, the medium spiny neurons (MSNs), which compose nearly 95% of the neurons in the NAc, are received projections from the majority of VTA DAergic neurons [8, 9]. Therefore, the NAc is closely involved in feeding-related motivation, suggesting that the NAc is a noteworthy target for treating a patient with anorexia.

A traditional Japanese (Kampo) medicine, ninjin’yoeito (NYT), which composed of 12 crude drugs (*Rehmanniae Radix*, *Angelicae Acutilobae Radix*, *Atractylodis Rhizoma*, *Poria*, *Ginseng Radix*, *Cinnamomi Cortex*, *Polygalae Radix*, *Paeoniae radix*, *Citri Unshiu Pericarpium*, *Astragali Radix*, *Glycyrrhizae Radix* and *Schisandrae Fruits*), is often prescribed in Japan for the treatment of declined constitution due to disease, fatigue and malaise, anorexia, perspiration during sleep, cold limbs, and anemia. Recently, NYT has been reported in clinical studies to ameliorate apathy, anorexia and cognitive dysfunction in patients with Alzheimer’s disease (AD) [8, 9]. Yamada et al. reported that NYT ameliorates decreased motivation of nest-building (as an indicator of goal-directed behavior) and amount of food intake (as an indicator of appetite) in mice with apathy-like behaviors induced by water immersion stress [10]. This report also showed that some ingredients of NYT inhibit DA transporter (DAT) and DA degrading enzymes, catechol-O-methyltransferase and monoamine oxidase inhibitor B, suggesting that NYT may regulate the DA transmission in the reward system. Since DAergic neurons project into areas such as the PFC, hippocampus, NAc and striatum [11], NYT may affect not only the DAergic neurons but also the postsynaptic neurons in these regions. Considering that NYT has no effect on impaired responding to food reward in the ventral striatal D2-MSN dysfunction mice [12], the ventral striatum including the NAc is one of the potential targets for NYT.

Thus, since NYT may have multiple effects on motivation-related functions (goal-directed behavior and feeding behavior), several brain regions related to the reward system are likely to be candidates for action sites of NYT. Motivation-related functions are controlled by DA signaling, which can be determined by the neuronal excitability changes of both presynaptic DAergic neurons and their postsynaptic neurons. Therefore, we considered that NYT may change the neuronal excitability of DAergic neurons in the VTA and SNpc, as well as that of MSNs in the NAc. However, it is unclear whether NYT can directly affect these neurons. In this study, in order to better characterize the mechanisms of NYT, we aimed to clarify the direct effects of NYT on VTA and SNpc DAergic neurons as presynaptic neurons, as well as NAc MSNs as postsynaptic neurons by using the patch-clamp technique in the brain slices.

## RESULTS

### Effects of ninjin’yoeito on the electrophysiological properties of VTA and SNpc DAergic neurons

We investigated the effect of NYT on the neuronal excitability of VTA and SNpc DAergic neurons in normal transgenic mice expressing tyrosine hydroxylase green fluorescence protein (TH-GFP) using the patch-clamp technique. Because Kampo medicines contain many pharmacological agents, it is difficult to determine the appropriate concentration for the experiments. Therefore, in order to clarify the appropriate concentration of NYT on DAergic neurons, we investigated the effects of low concentration NYT (0.1, 1 and 10 μg/mL) on VTA DAergic neurons. Low concentration NYT tends to decrease resting membrane potential (RMP), but the effects were not statistically significant (data not shown). However, as shown in this study, 100 μg/mL NYT showed a significant effect on DAergic neurons and thus we performed all the subsequent experiments in 100 μg/mL NYT.

**Figure 1:**
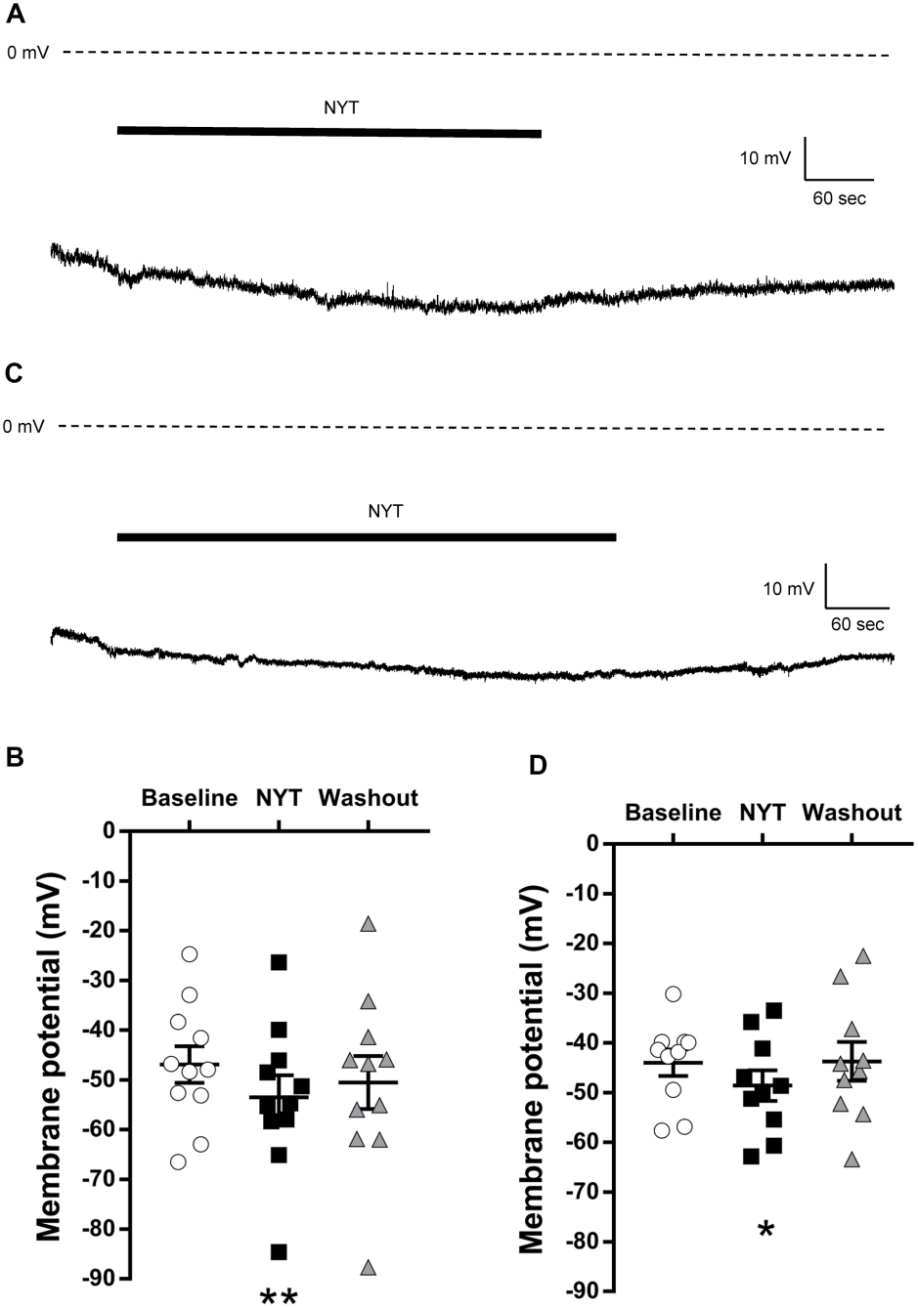
**The effects of NYT on the resting membrane potential of VTA and SNpc DAergic neurons.** (**A**) The representative resting membrane potential recording of a VTA DAergic neuron. (**B**) The effect of NYT on the resting membrane potential of VTA DAergic neurons (n = 11 from eight mice). (**C**) The representative resting membrane potential recording of an SNpc DAergic neuron. (**D**) The effect of NYT on the resting membrane potential of SNpc DAergic neurons (n = 10 from seven mice). Error bars are expressed as mean ± SEM. Statistical analyses were performed by one-way RM ANOVA followed by Dunnett’s multiple comparisons test, *p < 0.05, **p < 0.01; Baseline vs. NYT. The bars indicate the duration of the NYT application.

Figure 1A shows a representative RMP recording of a VTA DAergic neuron. NYT slightly but significantly reduced the RMP of VTA DAergic neurons (Figure 1B). A five-minute washout returned the membrane potential to the pre-application level. To clarify whether this is a VTA-specific change, we also examined the effects of NYT on DAergic neurons in the SNpc. Figure 1C shows a representative RMP recording of an SNpc DAergic neuron. NYT also slightly but significantly reduced the RMP of SNpc DAergic neurons (Figure 1D). This effect was also reversed after a 5-minute washout. Hyperpolarizing effect of NYT was also observed in DAergic neurons with spontaneous firing in the VTA and SNpc with a tendency to suppress firing frequency (Supplementary Figure 1).

These results show the possibility that NYT may not increase the neuronal excitability of the VTA and SNpc DAergic neurons.

### Effects of ninjin’yoeito on the electrophysiological properties of MSNs in the NAc core subregion

Since NYT failed to increase the neuronal excitability of VTA and SNpc DAergic neurons, we next investigated the electrophysiological effects of NYT on the neuronal excitability of NAc MSNs in normal C57BL/6J mice as postsynaptic neurons of DAergic neurons. Since the NAc region is further separated into two subregions (core and shell) [13–15], we examined the effects of NYT on MSNs in both subregions.

**Figure 2:**
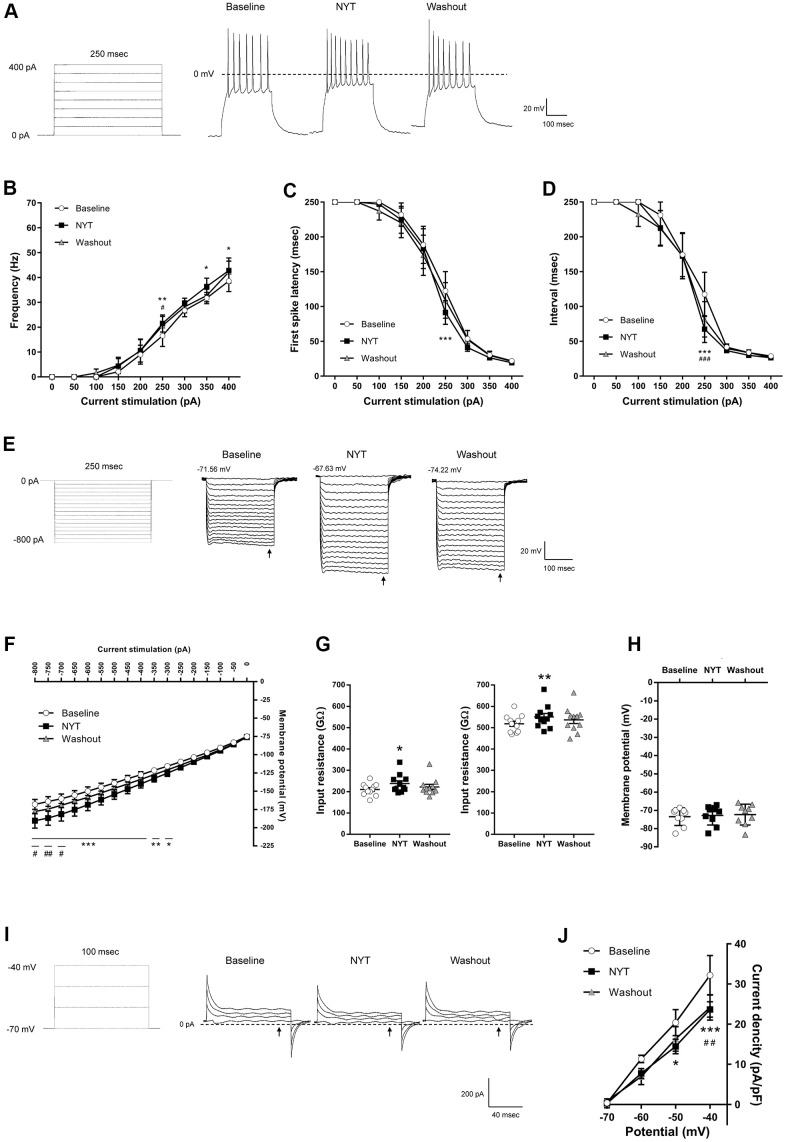
**The effects of NYT on the electrophysiological properties of MSNs in the NAc core subregion.** (**A**) The protocol of positive current injections (50 pA increments from 0 to 400 pA, 250 msec) and the representative membrane potential recordings obtained by 200 pA current injection. The relationship of (**B**) firing frequency, (**C**) first spike latency and (**D**) interspike interval obtained by positive current injections (n = 11 from seven mice). (**E**) The protocol of negative current injections (50 pA increments from -800 to 0 pA, 250 msec) and the representative membrane potential recordings obtained by negative current injections. (**F**) The I-V relationship obtained by negative current injections (n = 11 from seven mice). (**G**) The input resistance in -800 pA (left) and -200 pA (right) current injections (n = 11 from seven mice). (**H**) The resting membrane potential (n = 9 from seven mice). (**I**) The protocol of voltage pulses (10 mV increments from -70 to -40 mV, 100 msec, holding potential = -70 mV) and the representative current recordings obtained by voltage pulses. (**J**) The I-V relationship obtained by voltage pulses (n = 5 from two mice). Error bars are expressed as mean ± SEM. Statistical analyses were performed by one-way or two-way RM ANOVA followed by Dunnett’s multiple comparisons test, *p < 0.05, **p < 0.01, ***p < 0.001; Baseline vs. NYT, ^#^p < 0.05, ^##^p < 0.01, ^###^p < 0.001; NYT vs. Washout. The arrows indicate the time points of analysis.

Figure 2 shows the results of MSNs in the NAc core. Figure 2A shows a positive current stimulation protocol for the MSNs in the NAc core and its representative recordings obtained by 200 pA current injection. NYT significantly increased firing frequency in 250, 350 and 400 pA current injections compared to the baseline (Figure 2B). In addition, NYT significantly shortened the first spike latency and interspike interval (ISI) in 250 pA current injection compared to the baseline (Figure 2C, 2D). Figure 2E shows a negative current stimulation protocol for an inward rectification measurement and representative recordings obtained by the negative current injection. NYT significantly shifted the current-voltage (I-V) relationship downward between -800 and -300 pA current injections compared to the baseline (Figure 2F). The representative recording and the I-V relationship of NYT-sensitive membrane potential are shown in Supplementary Figure 2A. In a voltage-clamp mode experiment, NYT significantly inhibited inward currents between -110 and -100 mV voltage stimulation compared to the baseline (Supplementary Figure 2B, 2C). The inhibition is similar to the effect of inward rectifier potassium channel blocker, cesium chloride (CsCl) (Supplementary Figure 2D). In addition, NYT significantly raised the input resistance in -800 and -200 pA compared to the baseline (Figure 2G). In contrast, there was no significant effect of NYT on the RMP of MSNs in the NAc core (Figure 2H). Figure 2I shows a protocol of voltage stimulation for MSNs in the NAc core and representative recordings obtained by the voltage stimulation. NYT significantly shifted the I-V relationship downward between -50 and -40 mV voltage stimulation compared to the baseline (Figure 2J). The subtraction of the currents between baseline and after NYT application revealed the I-V relationship of NYT-sensitive outward currents (Supplementary Figure 2E). The effects of NYT on first spike latency and input resistance (-800 and -200 pA) were not detected after a 5-minute washout, but the other factors were not completely abolished after a 5-minute washout.

### Effects of ninjin’yoeito on the electrophysiological properties of MSNs in the NAc shell subregion

**Figure 3:**
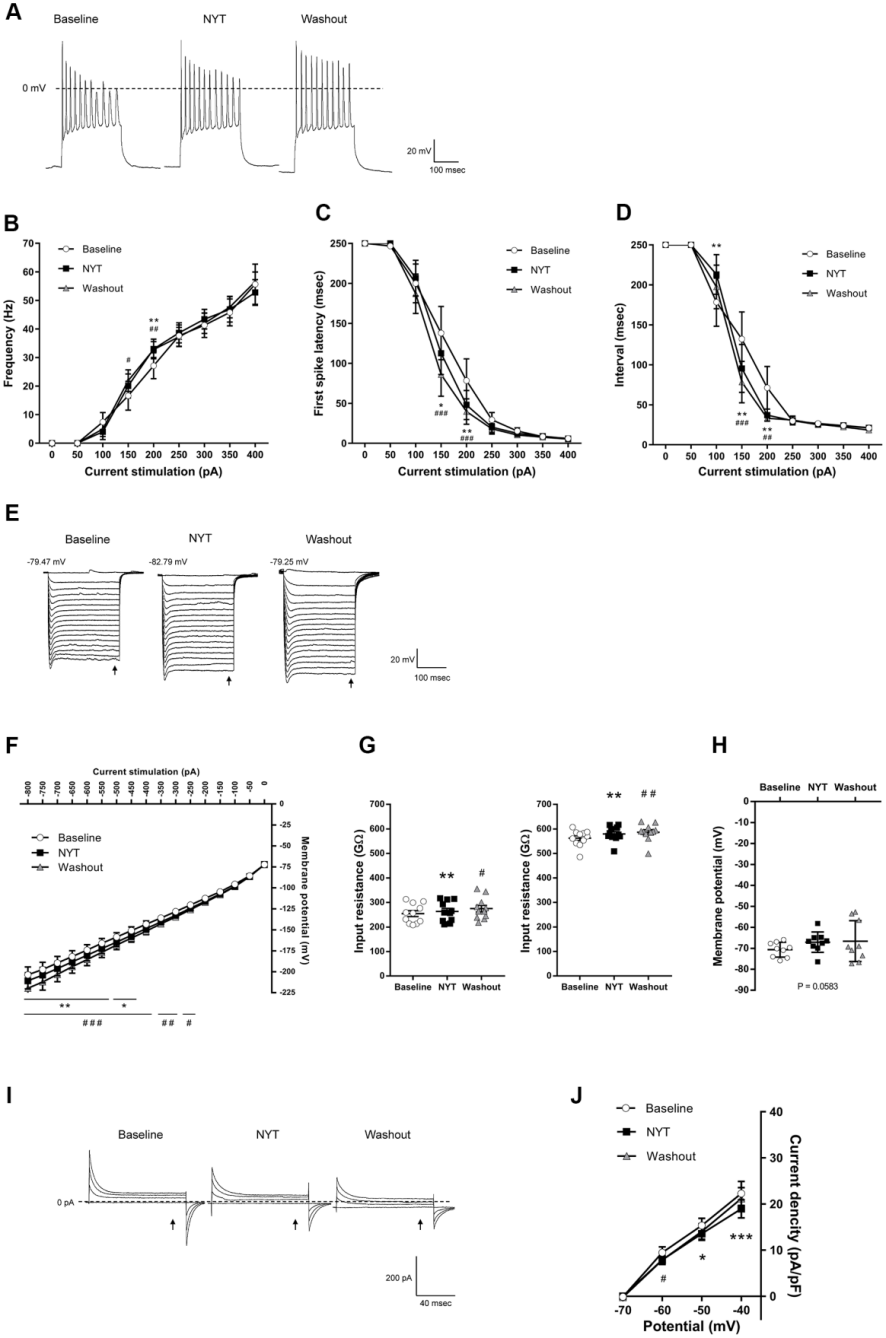
**The effects of NYT on the electrophysiological properties of MSNs in the NAc shell subregion.** (**A**) The representative membrane potential recordings obtained by 200 pA current injection. The relationship of (**B**) firing frequency, (**C**) first spike latency and (**D**) interspike interval obtained by positive current injections (n = 11 from nine mice). (**E**) The representative membrane potential recordings obtained by negative current injections. (**F**) The I-V relationship obtained by negative current injections (n = 11 from nine mice). (**G**) The input resistance in -800 pA (left) and -200 pA (right) current injections (n = 11 from nine mice). (**H**) The resting membrane potential (n = 9 from six mice). (**I**) The representative current recordings obtained by voltage pulses. (**J**) The I-V relationship obtained by voltage pulses (n = 6 from four mice). Error bars are expressed as mean ± SEM. Statistical analyses were performed by one-way or two-way RM ANOVA followed by Dunnett’s multiple comparisons test, *p < 0.05, **p < 0.01, ***p < 0.001; Baseline vs. NYT, ^#^p < 0.05, ^##^p < 0.01, ^###^p < 0.001; NYT vs. Washout. The arrows indicate the time points of analysis.

Figure 3 shows the results of MSNs of the NAc shell. Figure 3A shows representative recordings obtained by 200 pA current injection. NYT significantly increased firing frequency in 200 pA current stimulation compared to the baseline (Figure 3B), and significantly shortened first spike latency and ISI in 150 and 200 pA current injections compared to the baseline (Figure 3C, 3D). In contrast, ISI in 100 pA current stimulation was significantly lengthened by NYT. We obtained representative recordings by the negative current injection in the examination for inward rectification (Figure 3E). NYT significantly shifted the I-V relationship downward between -800 and -450 pA current injections compared to the baseline (Figure 3F). The representative recording and the I-V relationship of NYT-sensitive membrane potential are shown in Supplementary Figure 3A. In a voltage-clamp mode experiment, NYT significantly inhibited inward currents between -110 and -100 mV voltage stimulation compared to the baseline (Supplementary Figure 3B, 3C). The inhibition is similar to the effect of CsCl (Supplementary Figure 3D). In addition, NYT significantly raised the input resistance to -800 and -200 pA compared to the baseline (Figure 3G). In contrast, NYT tended to increase RMP compared to the baseline, unlike MSNs in the NAc core (Figure 3H). Figure 3I shows representative recordings by the voltage stimulation. NYT significantly shifted the I-V relationship downward between -50 and -40 mV voltage stimulation compared to the baseline (Figure 3J). The subtraction of the currents between baseline and after NYT application revealed the I-V relationship of NYT-sensitive outward currents (Supplementary Figure 3E). All of the effects of NYT were not completely disappeared by a 5-minute washout.

## DISCUSSION

In the present study, we demonstrated that NYT decreased the excitability of DAergic neurons in the VTA and SNpc, and enhanced the responsiveness to the current stimulation of MSNs in the NAc (core and shell). These results suggest that NYT may directly influence VTA and SNpc DAergic neurons and NAc MSNs.

Approximately 95% of the NAc neurons are composed of MSNs, which receive DAergic neuronal projections from the VTA [13, 15, 16], and are activated by DA neurotransmission [17, 18]. The NAc is morphologically, anatomically and functionally distinguished into core and shell subregions [13–15]. It is reported that core subregion mediates goal-directed behaviors such as spatial learning, reinforcement learning, responses to conditioned and motivational stimulation, and effortful decision-making, whereas shell subregion mediates encoding the value of stimulations such as processing hedonic or reward value, responding to novelty, and feeding [13, 15]. Since previous studies have indicated that NYT has positive effects on anorexia, decreased goal-directed behaviors and anhedonia-like traits [8, 10, 12], we investigated the effects of NYT on neuronal excitability in both the NAc core and shell.

NYT increased the frequency of firings induced by the current injections in NAc (core and shell) MSNs (Figures 2B, 3B). In addition, we show that sustained outward currents of NAc MSNs (core and shell) were suppressed by NYT (Figures 2I, 2J, 3I, 3J). Although there are few reports regarding the sustained outward currents, previous studies suggested that inhibition of delayed rectifiers and BK channels, both of which mediate sustained outward currents, is responsible for the increase in firing frequency [18–20]. In particular, suppression of sustained outward currents mediated by delayed rectifiers has been reported to shorten the first spike latency [20], which is similar to our results (Figures 2C, 3C). The increase of input resistance by NYT application (Figures 2G, 3G) suggests the blockage of some channels including delayed rectifiers or BK channels. Some ingredients within NYT (paeonol, nobiletin, 18β-glycyrrhetinic acid) have been suggested to inhibit sustained outward currents (mediated by TEA-sensitive channels, BK channels, Kv1.3, and Kv2.1) [21–24]. Similarly, DA is known to decrease the sustained outward currents via activation of D1 receptors, resulting in depolarization of membrane potential and increasing the firing frequency of NAc MSNs [18, 20, 25]. Therefore, inhibition of sustained outward currents by NYT (or its ingredients) may enhance the response to DA signaling in NAc MSNs.

NYT shifted the I-V curve downward for negative current injections (Figures 2F, 3F). In addition, inward currents in the voltage-clamp experiments were decreased by NYT (Supplementary Figures 2B, 2C, 3B, 3C). These results indicate that NYT may attenuate the inward rectification, which is characteristic in NAc MSNs [26–30], suggesting that NYT may block the inward-rectifying potassium currents. Since classic inward rectifier potassium channels and G-protein-activated inward rectifiers, both of which mediate inward-rectifying potassium currents, are expressed in the NAc [31, 32], NYT may block these potassium channels. Several previous studies have reported that DA reduces inward rectification via D1 and D2 receptor activation, which can increase the cellular excitability of NAc MSNs, resulting in increased firing frequency [19, 33, 34]. Hence, inhibition of potassium channels mediating inward-rectifying currents by NYT may enhance DA signaling.

The NAc (core and shell) MSNs project to the globus pallidus, VTA, SNpc, and lateral hypothalamus, whose projections regulate feeding behavior [7, 35, 36]. DA is released during various responses to rewards (in a moment predicting rewards by relying on cues, acting to obtain rewards, consuming rewards, and reacting to unexpected rewards) in the NAc [1, 5, 7, 13, 35, 36]. Specific ablation of dopamine in the NAc has been reported to attenuate goal-directed behavior regarding food [37, 38]. Moreover, the administration of D1 antagonist in the NAc core impairs motivational behaviors for food rewards [39]. Mice lacking D2 receptors show decreased food intake and impaired reward-related motivation [40, 41]. Conversely, optogenetic activation of the D2 receptor-expressing MSNs in the NAc increases motivation for food rewards [42–44]. Administration of indirect DA agonist amphetamine into the NAc shell induces palatable food motivation [45]. Thus, DA signaling in the NAc plays a critical role in the motivational regulation of food. Yamada et al. reported that NYT reverses decreased motivation for nest-building and reduction of food intake in mice with apathy-like behaviors induced by water immersion stress, whose effects may be exerted through D2 receptor activation [10]. The similarity of the effects on the potassium channels between DA and NYT found in the present study indicates the possibility that NYT may enhance the responsiveness of NAc MSNs to DA signaling, suggesting that NYT may increase motivation for food and goal-directed behavior.

On the other hand, it should be noted whether activation of NAc MSNs and DA signaling leads to promotion or inhibition of reward-related behaviors remains controversial [45–48]. These inconsistent reports may be due to the influence such as the heterogeneous spread of NAc neurons with distinct characters, the spatial gradient of expression of D1/D2 receptors in the NAc, and the environments in which the individual is placed [48–50]. Although NYT improves anorexia and decreased motivation in AD patients in the clinical study [8], the characteristics of the NAc MSNs in AD patients are required to be revealed to determine if the NAc and DA signaling can be involved in the underlying mechanism of the clinical improvement by NYT.

The NAc interacts not only with the VTA and SNpc but also with the PFC and hippocampus. Therefore, the NAc has been reported to be implicated in numerous neurological and psychiatric disorders including AD [15]. Some reports have revealed that the interactions of the NAc to the PFC and/or hippocampus may be implicated in spatial cognitive function [51–53]. In addition, atrophy of the NAc is associated with cognitive impairment in AD patients [54]. Indeed, since it is reported that NYT ameliorates cognitive dysfunction in AD patients [8, 9], the increased neuronal excitability of NAc MSNs by NYT could contribute to the improvement of cognitive dysfunction.

Considering the possibility that NYT improved anorexia and impaired motivation by directly affecting DAergic neurons, we investigated the effects of NYT on DAergic neurons in the VTA and SNpc. Our results showed that NYT reduced the RMP of DAergic neurons in the VTA and SNpc (Figure 1). Although we investigated the involvement of D2 autoreceptors of DAergic neurons (Supplementary Figure 4) and inhibitory postsynaptic currents (IPSCs) from GABAergic interneurons (Supplementary Figure 5), which are abundant in the VTA region, as the cause of the RMP reduction in the VTA, no effect was observed. This implies that there are other factors rather than D2 autoreceptors and IPSCs for the RMP lowering effect of NYT. Hesperetin, an ingredient of Citrus Unshiu Peel (one of the components of NYT), has been reported to activate opioid receptors [55, 56]. Activation of opioid receptors expressed in VTA and SNpc DAergic neurons can reduce the RMP and firing rate [57, 58]. Moreover, in a previous report, Oizumi et al. also suggested that the prevention of anhedonia-like traits by NYT could be induced by the participation of opioid signaling [12]. Therefore, activation of opioid receptors by hesperetin in NYT may contribute to the decreased RMP of DAergic neurons. Although other ingredients (formononetin, isoliquiritigenin, paeonol) are reported to activate BK channels or Kv1.2 [59, 60], there is no evidence that these channels of DAergic neurons are involved in regulating RMP.

Psychostimulants such as cocaine and methamphetamine are widely known as drugs acting on DAergic neurons. Especially, methamphetamine not only blocks DAT but also promotes the firing frequency of DAergic neurons [61]. Although NYT has also been suggested to block DAT [10], the results of the present study (Figure 1) suggest that NYT may not excite DAergic neurons in the VTA and SNpc abnormally. Therefore, NYT is likely to have different characteristics from conventional psychotropic drugs acting on the DAergic pathway. Similar to the effects on DAergic neurons, NYT did not depolarize the RMP of NAc MSNs (Figures 2H, 3H). These results indicate that NYT may not induce abnormal excitation in NAc MSNs as well as DAergic neurons in a normal condition, suggesting that NYT may modulate signaling from presynaptic neurons (such as DA neurons) by altering the responsiveness of the postsynaptic neurons in the NAc.

In conclusion, we reveal that NYT may directly influence the excitability of DAergic neurons in the VTA and SNpc, as well as MSNs in the NAc (core and shell) by investigating the effects of NYT on the electrophysiological properties of the neurons that comprise the reward system in this study. We show that NYT decreased the neuronal excitability of VTA and SNpc DAergic neurons. In contrast, we also demonstrate that NYT increased the frequency of firing induced by current stimulation of NAc MSNs (core and shell), which may be due to the inhibition of sustained outward potassium currents and inward-rectifying potassium currents. Considering that DA also inhibits these currents via activation of D1 and D2 receptors, the inhibition of these currents by NYT may partly mimic a DA transmission. This indicates that NYT may work as a DA signaling modulator in the NAc. NYT may contain both excitatory and inhibitory ingredients for the neurons, suggesting that NYT could regulate the DAergic pathway according to physiological conditions. In this study, we conducted experiments only with NYT extract that include multi-ingredient. However, experiments using ingredients in NYT are also necessary to fully explain the action mechanism of NYT. Further single-ingredient studies may reveal the target molecules, which can clarify whether NYT influences neurons by acting directly on ion channels or indirectly through other receptors. Although this study is the first report to show that NYT has effects on VTA and SNpc DAergic neurons, as well as NAc MSNs, further studies of NYT ingredients are required to clarify its pharmacological action. Our findings provide us with an explanation of the action mechanism of NYT in improving anorexia and decreased motivation.

## MATERIALS AND METHODS

### Animals

Male C57BL/6J mice were purchased from The Jackson Laboratory Japan, Inc. (Yokohama, Japan). Male TH-GFP-expressing transgenic mice were obtained from the Institute of Physical and Chemical Research (RIKEN BRC, Ibaraki, Japan) [62]. C57BL/6J mice (aged 4–8 weeks old) and transgenic mice expressing TH-GFP (aged 7–34 weeks old) were used in experiments. C57BL/6J mice and TH-GFP-expressing transgenic mice were housed in a 12-hour light/dark cycle (07:00–19:00) with conventional food (MF, Oriental Yeast Co., Ltd., Tokyo, Japan; CE-2, CLEA, Tokyo, Japan) and water *ad libitum*. Animal experiments with C57BL/6J mice were approved by the Experimental Animal Ethics Committees of Tsumura & Co. and animal experiments with TH-GFP-expressing transgenic mice were approved by the Institutional Animal Care and Use Committee of Fukushima Medical University.

### Preparation of brain slices

C57BL/6J mice were used for the experiments targeting NAc MSNs and TH-GFP-expressing transgenic mice were used for the experiments targeting VTA and SNpc DAergic neurons. The mice were transcardially perfused with an ice-cold solution containing (in mM) 230 sucrose, 2 KCl, 1 KH_2_PO_4_, 0.5 CaCl_2_, 1 MgCl_2_, 26 NaHCO_3_, and 10 D-glucose under anesthesia, followed by isolation of the whole brain. In the ice-cold solution, coronal brain slices (200 μm thick) including the NAc (approximately 0.7 mm to 1.7 mm from the bregma) or VTA and SNpc (approximately -3.2 mm to -2.8 mm from the bregma) [63] were prepared using a microtome (Campden Instruments, Leics., UK). The slices were recovered for 1 h or more in room temperature artificial cerebrospinal fluid (aCSF) containing (in mM) 126 NaCl, 2.5 KCl, 1.2 MgCl_2_, 2.4 CaCl_2_, 1.2 NaH_2_PO_4_, 21.4 NaHCO_3_, and 10 D-glucose, with a gas mixture of 95% O_2_ and 5% CO_2_.

### Electrophysiology

The brain slices were transferred to a recording chamber. The gassed aCSF containing 0.1% dimethyl sulfoxide (DMSO) was perfused continuously at 2–4 mL/min. Whole-cell recordings were performed using an EPC-800 patch-clamp amplifier (HEKA Electronics, Lambrecht/Pfalz, Germany) filtered at 0.7 kHz for IPSC recordings or 1 kHz for the other recordings. The data were digitized with an analog-to-digital converter (Molecular Devices, CA, USA) and stored on a computer using Clampex 10 software (Molecular Devices). Patch electrodes (5–11 MΩ) were filled with an internal solution containing (in mM) 120 K-gluconate, 10 KCl, 10 HEPES, 5 EGTA, 0.3 CaCl_2_, 1 MgCl_2_, 2 Mg-ATP, and 1 Na-GTP at pH 7.3 adjusted with KOH. The electrophysiological experiments were performed at room temperature (21–27° C). Whole-cell patch recordings were performed for all recordings. Once the whole-cell configuration was acquired, all records were initiated after the stabilization period. Drugs were applied to each brain slice via bath perfusion.

The core and shell subregion of the NAc were identified with the anterior commissure and lateral ventricle as landmarks based on the mouse brain atlas [63]. Before the evaluation of NYT, NAc MSNs were identified by the following characteristics: medium-sized soma, inward rectification, a slow-ramping subthreshold depolarization in response to low-magnitude positive current injection, prominent spike afterhyperpolarization and/or an RMP lower than -65 mV [26–29]. VTA and SNpc DAergic neurons of transgenic mice expressing TH-GFP were identified with GFP fluorescence under a fluorescence microscope.

### Data recordings and analysis for VTA and SNpc DAergic neurons

All data were analyzed with clampfit software 10 (Molecular Devices). The membrane potential was recorded in a current-clamp mode.

In the RMP recordings, we compared the RMP at baseline (for 1 minute before NYT application), after 2-minute NYT treatment, and after a 5-minute washout. We compared the RMP after sulpiride application (for 1 minute before additional NYT application) and after 1-minute treatment of NYT + sulpiride. The firing frequency was compared between baseline (for 1 minute before NYT application) and after 1-minute treatment of NYT.

The IPSCs with -60 mV holding voltage in a voltage-clamp mode were recorded in the presence of 20 μM 6-cyano-7-nitroquinoxaline-2,3-dione disodium salt (CNQX) and 50 μM D-(−)-2-amino-5-phosphonopentanoic acid (AP5). Only > 5 pA events were accepted for IPSC analysis. The frequency and amplitude of IPSCs were compared between baseline (for 1 minute before NYT application) and after 1-minute treatment of NYT. The IPSCs were identified by applying 100 μM picrotoxin after each recording.

### Data recordings and analysis for NAc MSNs

All data were analyzed with clampfit software 10 (Molecular Devices). The membrane potential was recorded in a current-clamp mode.

A frequency-current relationship was obtained by positive current stimulation from 0 pA to 400 pA in increments of 50 pA steps as described previously, but with a minor modification [19]. The first spike latency was measured as the time from the onset of current stimulation to the first action potential peak. The ISI was measured as the time between action potentials. In the case of recordings without action potentials, 250 msec (duration of current stimulation) was assigned for the first spike latency and the ISI. The input resistance was measured by 250 msec negative currents from -800 to 0 pA in increments of 50 pA, as described previously [19]. The I-V relationship was analyzed at 230 msec after the initial current injection. Input resistance in the non-rectified range was calculated from the membrane potential in response to -200 pA hyperpolarizing pulses and input resistance in the rectified range was calculated from the membrane potential in response to the most hyperpolarizing current pulse (-800 pA) injected into the MSNs [29]. The membrane potential was recorded before NYT treatment (baseline), after 5–15-minute NYT treatment, and after a 5-minute washout. NYT-sensitive membrane potential was isolated by subtraction of the after-NYT membrane potential from the before-NYT membrane potential.

For the measurement of currents in voltage-clamp mode, the cells were held at -70 mV. The inward currents were evoked by 100 msec voltage depolarization to voltage values between -110 and -70 mV in 10 mV increments. The outward currents were evoked by 100 msec voltage depolarization to voltage values between -70 and -40 mV in 10 mV increments. The amplitude of the sustained outward currents was analyzed at 85 msec from initial voltage stimulation. Each current was calculated by subtracting the leak-current of each voltage step. The currents were recorded before NYT treatment (baseline), after 5–15-minute NYT treatment, and after a 5-minute washout. Also, the currents were recorded before CsCl treatment (baseline), after 3–15-minute CsCl treatment. NYT-sensitive currents were isolated by subtraction of the after-NYT current from the before-NYT current. These currents were also calculated by subtracting the leak-current of each voltage step.

### Drugs

NYT (Lot No. 362113100 and 372176700) was obtained from Tsumura & Co. (Tokyo, Japan), manufactured by spray-drying a hot water extract of a mixture of 12 crude drugs: The Japanese Pharmacopoeia (JP) Rehmannia Root (4.0 g), JP Japanese Angelica Root (4.0 g), JP Atractylodes Rhizome (4.0 g), JP Poria Sclerotium (4.0 g), JP Ginseng (3.0 g), JP Cinnamon Bark (2.5 g), JP Polygala Root (2.0 g), JP Peony Root (2.0 g), JP Citrus Unshiu Peel (2.0 g), JP Astragalus Root (1.5 g), JP Glycyrrhiza (1.0 g) and JP Schisandra Fruit (1.0 g). Plant materials were authenticated by identification of external morphology and marker compounds for plant specimens according to the methods of the Japanese Pharmacopeia and company standards. Extract quality was standardized based on the good manufacturing practice as defined by the Ministry of Health, Labour, and Welfare of Japan. NYT (0.1, 1, 100 μg/mL) was suspended in aCSF (including 0.1% DMSO) and used after filtering (0.22 μm) in the electrophysiological experiments. Sulpiride (D2 receptor antagonist, 1 μM), CNQX (20 μM), AP5 (50 μM) and CsCl (1 mM) were dissolved in aCSF (including 0.1% DMSO) in the electrophysiological experiments.

### Statistical analysis

All data were expressed as mean ± SEM. All statistical analysis was performed using Prism 7 (GraphPad, San Diego, California, USA). The spike frequency, first spike latency, ISI, membrane potential in negative current injection and amplitude of inward/outward currents (in the experiments of NAc MSNs) were analyzed using two-way repeated-measures (RM) analysis of variance (ANOVA) followed by Dunnett’s multiple comparisons test. The input resistance (in the experiments of NAc) and RMP (in the experiments of NAc, VTA and SNpc) were analyzed using one-way RM ANOVA followed by Dunnett’s multiple comparisons test. The RMP (in the experiment using sulpiride), as well as frequency and amplitude of IPSCs were analyzed using paired *t*-test. The firing frequency of VTA and SNpc DAergic neurons was analyzed using one-way RM ANOVA followed by Dunnett’s multiple comparisons test or paired *t*-test. The inward currents (in the experiment using CsCl) were analyzed using two-way RM ANOVA followed by Bonferroni’s multiple comparisons test. A value of P < 0.05 was considered to be statistically significant.

## Supplementary Material

Supplementary Figures
