# Supplementary material for: The effects of ninjin’yoeito on the electrophysiological properties of dopamine neurons in the ventral tegmental area/substantia nigra pars compacta and medium spiny neurons in the nucleus accumbens
Source: Aging (Albany NY). 2022 Jun 3;14(11):4634–52. doi: 10.18632/aging.204109 (PMC9217710; doi:10.18632/aging.204109)
Supplement: Supplementary Figures [file aging-14-204109-s001.pdf]

SUPPLEMENTARY FIGURES

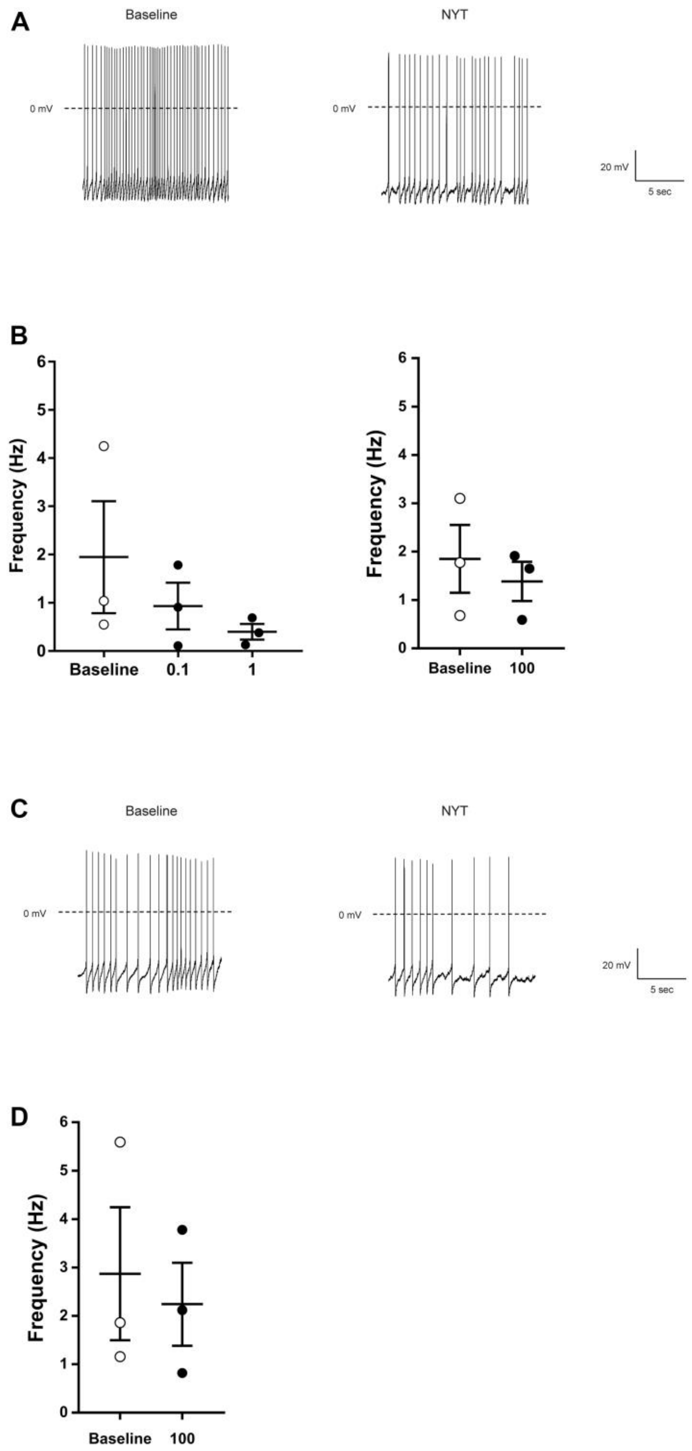

Supplementary Figure 1. The effects of NYT on the firing DAergic neurons in the VTA and SNpc. (**A**) The representative membrane potential recordings of firing DAergic neuron in the VTA before and after NYT (100 μg/mL) application. (**B**) The effect of NYT (0.1, 1, 100 μg/mL) on the firing frequency of VTA DAergic neurons (n = 3 from three mice). (**C**) The representative membrane potential recordings of the firing DAergic neuron in the SNpc before and after NYT (100 μg/mL) application. (**D**) The effect of NYT (100 μg/mL) on the firing frequency of SNpc DAergic neurons (n = 3 from three mice). Error bars are expressed as mean ± SEM. Statistical analyses were performed by (**B** (left)) one-way RM ANOVA followed by Dunnett's multiple comparisons test and (**B** (right) and **D**) paired *t*-test.

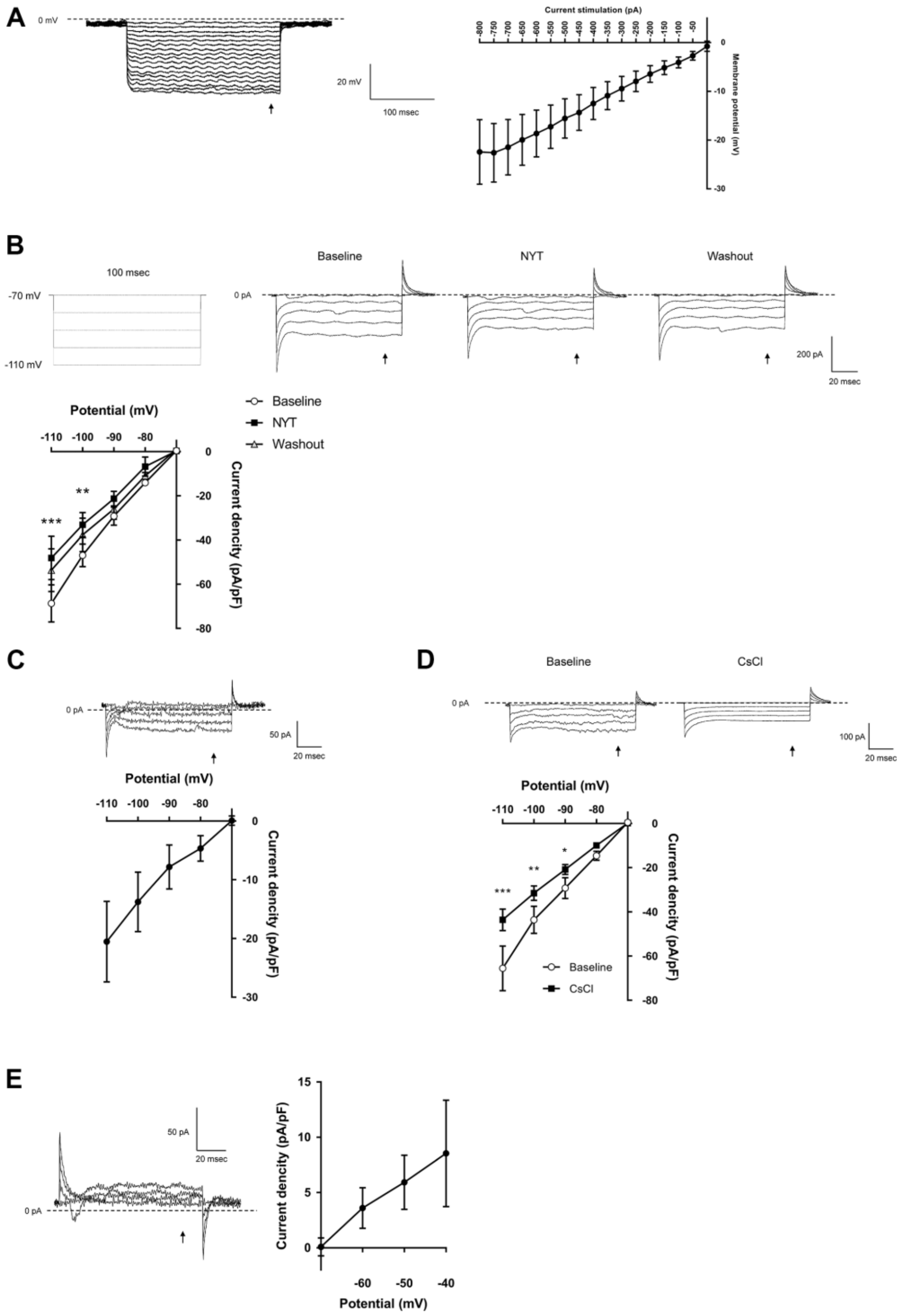

Supplementary Figure 2. The effects of NYT on the electrophysiological properties of MSNs in the NAc core subregion. (**A**) The representative NYT-sensitive membrane potential recording and the I-V relationship of NYT-sensitive membrane potential (n = 11 from seven mice). (**B**) The protocol of voltage pulses (10 mV increments from -110 to -70 mV, 100 msec, holding potential = -70 mV) for inward currents and the representative current recordings obtained by voltage pulses. The I-V relationship obtained by the voltage pulses (n = 5 from two mice). (**C**) The representative NYT-sensitive inward current recording and the I-V relationship of NYT-sensitive currents by voltage pulses (n = 5 from two mice). (**D**) The representative inward current recordings before and after CsCl application and the I-V relationship obtained by the voltage pulses (n = 4 from four mice). (**E**) The representative NYT-sensitive outward current recording and I-V relationship obtained by voltage pulses (n = 5 from two mice). The NYT-sensitive membrane potential and inward/outward currents were obtained by subtracting the trace after NYT application from the trace of baseline. Error bars are expressed as mean ± SEM. Statistical analyses were performed by (**B**) two-way RM ANOVA followed by Dunnett's multiple comparisons test (**$p < 0.01$, ***$p < 0.001$; Baseline vs. NYT) or (**D**) two-way RM ANOVA followed by Bonferroni's multiple comparisons test (*$p < 0.05$, **$p < 0.01$, ***$p < 0.001$; Baseline vs. CsCl). The arrows indicate the time points of analysis.

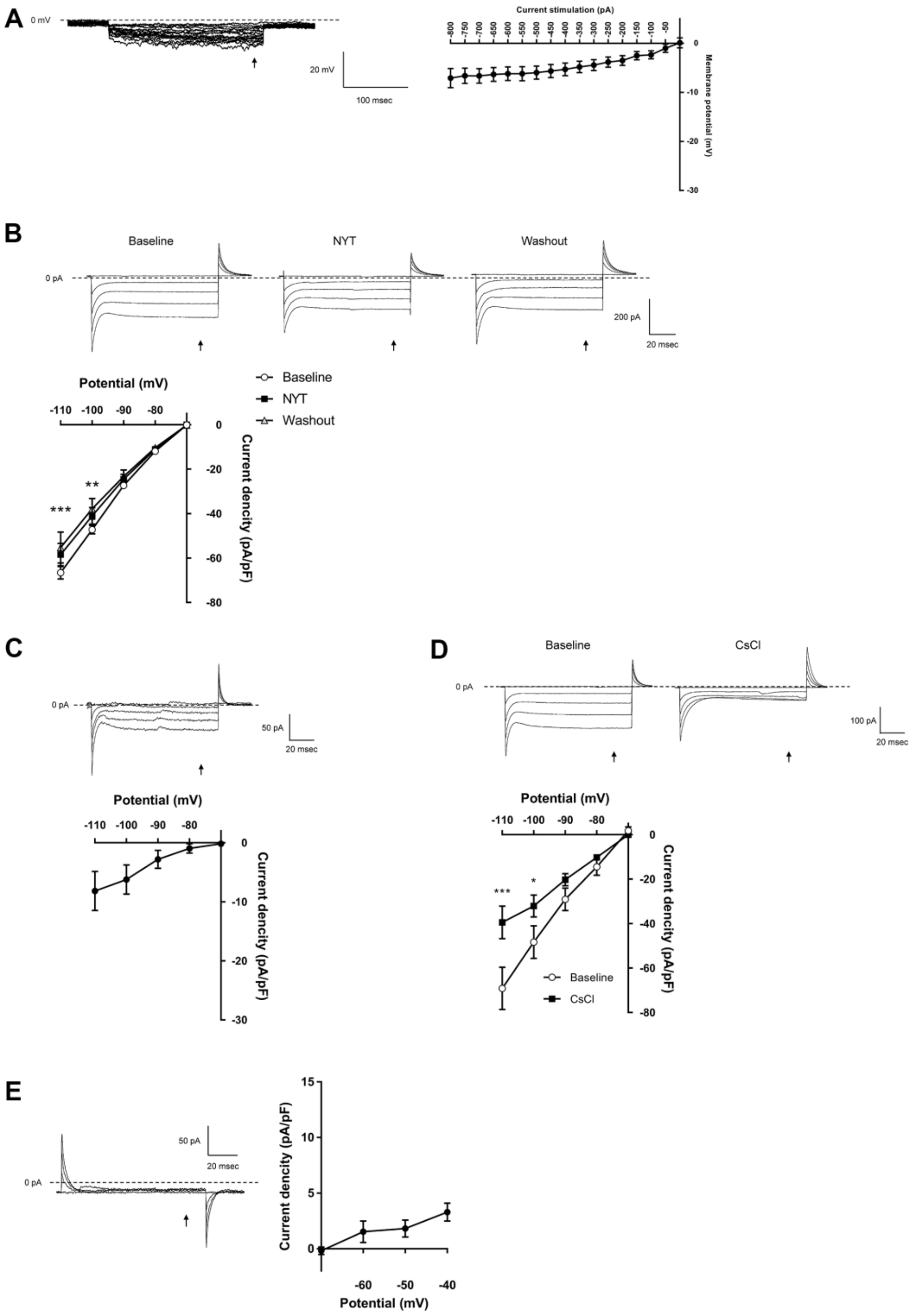

Supplementary Figure 3. The effects of NYT on the electrophysiological properties of MSNs in the NAc shell subregion. (**A**) The representative NYT-sensitive membrane potential recording and the I-V relationship of NYT-sensitive membrane potential (n = 11 from nine mice). (**B**) The representative current recordings and the I-V relationship obtained by voltage pulses (n = 6 from four mice). (**C**) The representative NYT-sensitive inward current recording and the I-V relationship by voltage pulses (n = 6 from four mice). (**D**) The representative inward current recordings before and after CsCl application and the I-V relationship obtained by voltage pulses (n = 4 from 3 mice). (**E**) The representative NYT-sensitive outward current recording and I-V relationship obtained by voltage pulses (n = 6 from four mice). The NYT-sensitive membrane potential and inward/outward currents were obtained by subtracting the trace after NYT application from the trace of baseline. Error bars are expressed as mean ± SEM. Statistical analyses were performed by (**B**) two-way RM ANOVA followed by Dunnett's multiple comparisons test (**p < 0.01, ***p < 0.001; Baseline vs. NYT) or (**D**) two-way RM ANOVA followed by Bonferroni's multiple comparisons test (*p < 0.05, ***p < 0.001; Baseline vs. CsCl). The arrows indicate the time points of analysis.

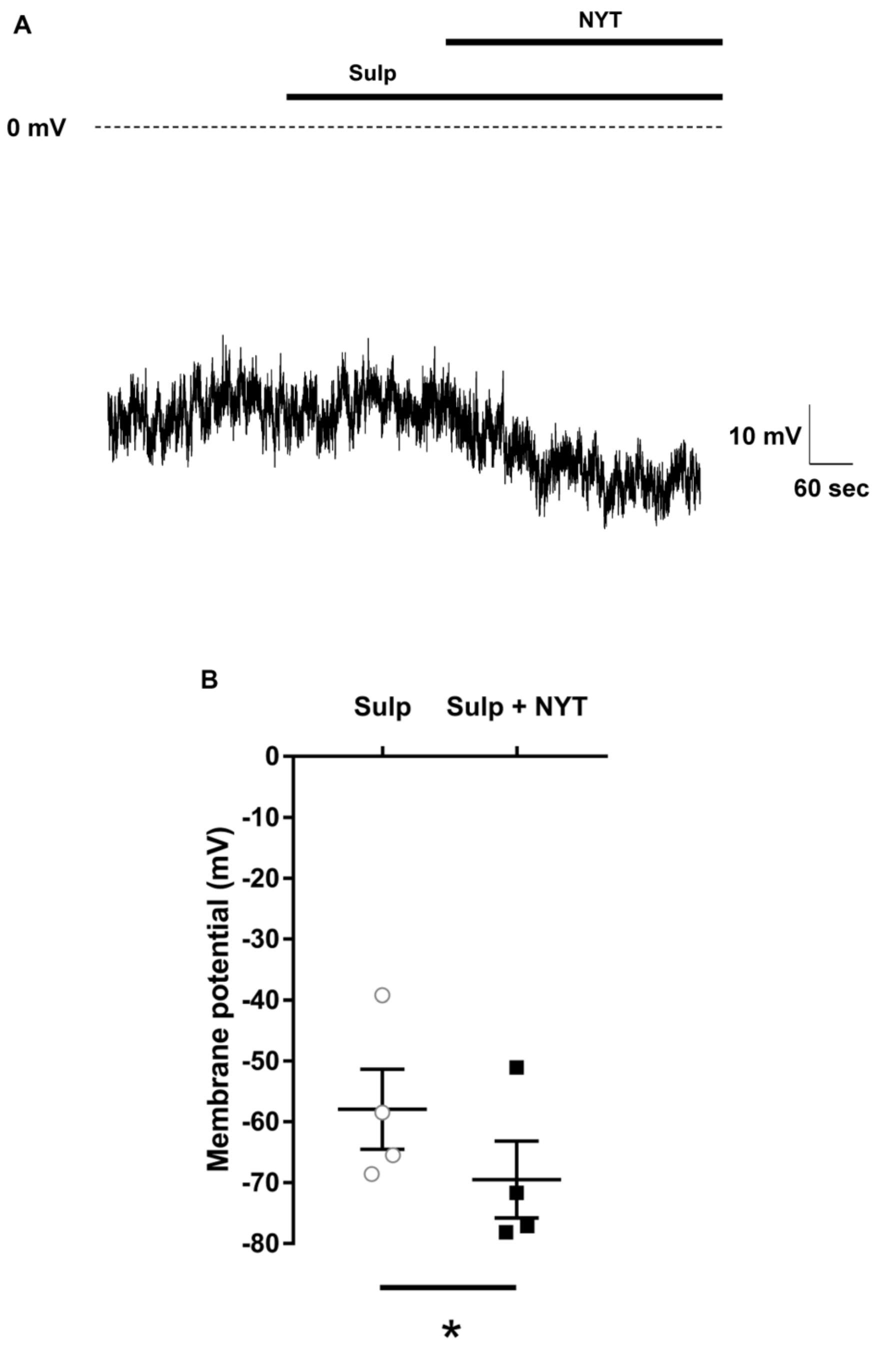

Supplementary Figure 4. The effects of NYT on dopamine D2 autoreceptor of VTA DAergic neurons. (**A**) The representative resting membrane potential recording of VTA DAergic neuron after sulpiride and NYT application. The bars indicate the duration of sulpiride or NYT application. (**B**) The resting membrane potential of VTA DAergic neurons after sulpiride and NYT application in TH-GFP mice. $^{*}p < 0.05$; sulpiride vs. sulpiride + NYT (n = 4 from three mice, paired *t*-test). Error bars are expressed as mean ± SEM.

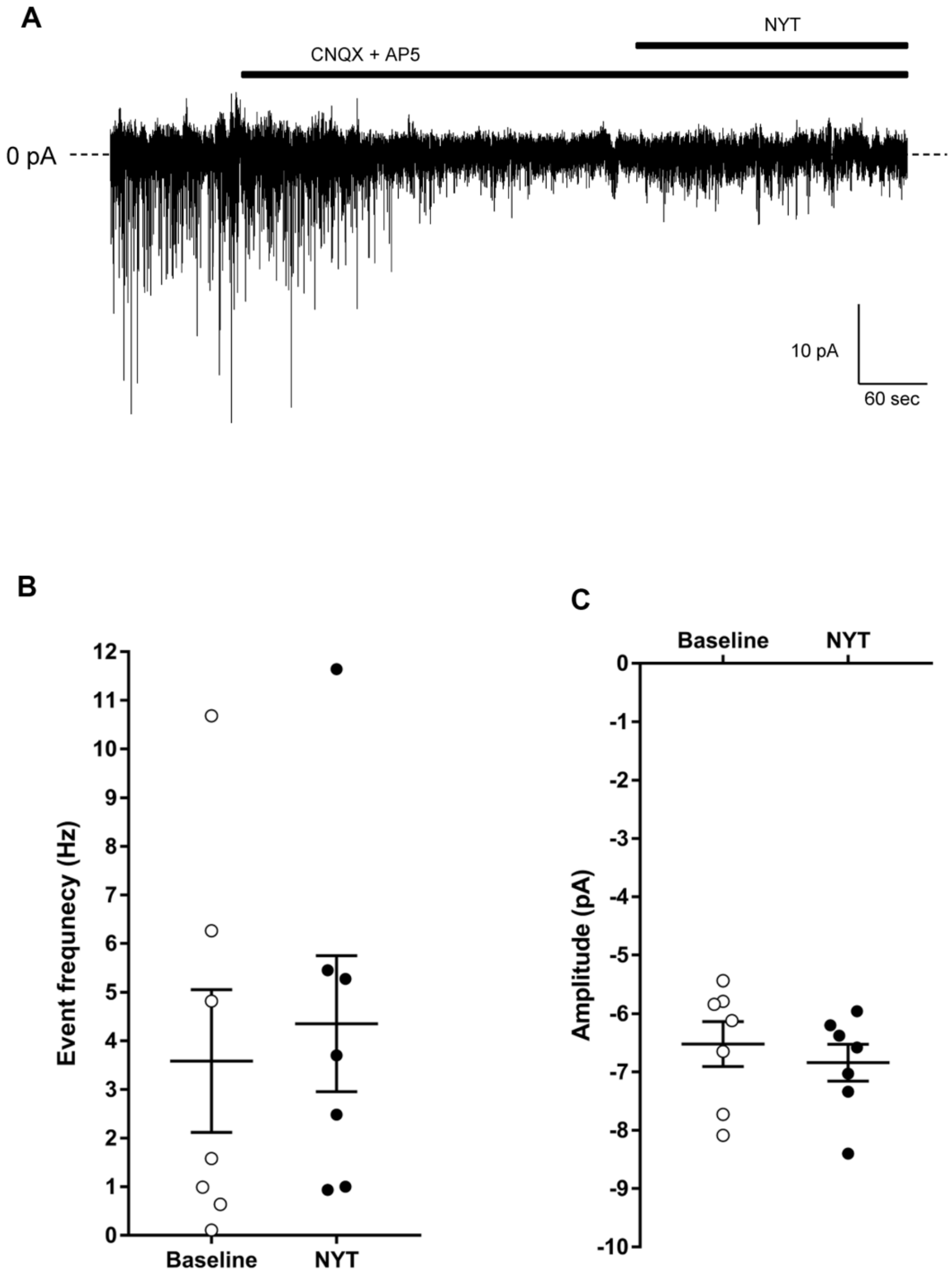

Supplementary Figure 5. The effects of NYT on IPSCs of VTA DAergic neurons. (**A**) The representative IPSC recording of VTA DAergic neuron after NYT application. The bars indicate the duration of CNQX, AP5 or NYT application. (**B**) Frequency and (**C**) amplitude of IPSC inputs into the VTA DAergic neurons after NYT application. (n = 7 from three mice, paired *t*-test). Error bars are expressed as mean ± SEM.
